# (18-Crown-6)(trifluoro­methane­sulfonato)­sodium

**DOI:** 10.1107/S1600536810025961

**Published:** 2010-07-07

**Authors:** Hien Ngoc Phan, Hans-Wolfram Lerner, Michael Bolte

**Affiliations:** a Institut für Anorganische Chemie, J. W. Goethe-Universität Frankfurt, Max-von-Laue-Strasse 7, 60438 Frankfurt/Main, Germany

## Abstract

The title compound, [Na(CF_3_O_3_S)(C_12_H_24_O_6_)], features a sodium cation that is coordinated by eight O atoms in an irregular hexa­gonal bipyramidal environment. The equatorial positions are occupied by the six O atoms of an 18-crown-6 ether ring. In the axial positions, there is one O atom of a trifluoro­methane­sulfonate anion and an ether O atom of a symmetry-equivalent crown ether ring. In this way, centrosymmetric dimers are formed.

## Related literature

For the synthesis of hetereoleptic transition metal complexes with silyl ligands, see: Lerner (2005 ▶). For the reaction of Na_2_[Fe(CO)_4_] with *t*Bu_3_SiO_3_SCF_3_, see: Lerner *et al.* (2002 ▶). For the structure of similar complexes with trifluoro­methane­sulfonate, see: Bolte & Lerner (2001 ▶); Lerner & Bolte (2003 ▶); Sofina *et al.* (2003 ▶); Dinnebier *et al.* (2004 ▶); Hilde­brandt *et al.* (2006 ▶).
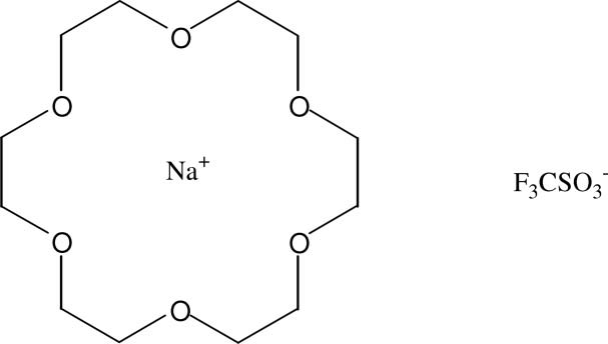


## Experimental

### 

#### Crystal data

[Na(CF_3_O_3_S)(C_12_H_24_O_6_)]
*M* *_r_* = 436.37
Monoclinic, 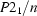

*a* = 9.4455 (9) Å
*b* = 15.1723 (12) Å
*c* = 14.0597 (14) Å
β = 100.828 (8)°
*V* = 1979.0 (3) Å^3^
*Z* = 4
Mo *K*α radiation
μ = 0.26 mm^−1^
*T* = 173 K
0.33 × 0.20 × 0.19 mm

#### Data collection

Stoe IPDS II two-circle diffractometer
Absorption correction: multi-scan (*MULABS*; Spek, 2009 ▶; Blessing, 1995 ▶) *T*_min_ = 0.921, *T*_max_ = 0.943
11867 measured reflections
3697 independent reflections
2856 reflections with *I* > 2σ(*I*)
*R*_int_ = 0.039

#### Refinement

*R*[*F* ^2^ > 2σ(*F* ^2^)] = 0.044
*wR*(*F* ^2^) = 0.111
*S* = 1.03
3697 reflections
245 parameters
H-atom parameters constrained
Δρ_max_ = 0.55 e Å^−3^
Δρ_min_ = −0.44 e Å^−3^

### 

Data collection: *X-AREA* (Stoe & Cie, 2001 ▶); cell refinement: *X-AREA*; data reduction: *X-AREA*; program(s) used to solve structure: *SHELXS97* (Sheldrick, 2008 ▶); program(s) used to refine structure: *SHELXL97* (Sheldrick, 2008 ▶); molecular graphics: *XP* (Sheldrick, 2008 ▶); software used to prepare material for publication: *SHELXL97*.

## Supplementary Material

Crystal structure: contains datablocks I, global. DOI: 10.1107/S1600536810025961/ng2797sup1.cif

Structure factors: contains datablocks I. DOI: 10.1107/S1600536810025961/ng2797Isup2.hkl

Additional supplementary materials:  crystallographic information; 3D view; checkCIF report

## Figures and Tables

**Fig. 1.**
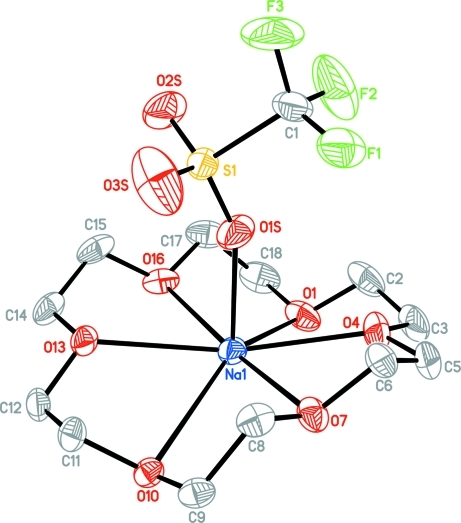
Perspective view of the title compound with the atom numbering scheme; displacement ellipsoids are at the 50% probability level; H atoms are omitted for clarity.

**Fig. 2.**
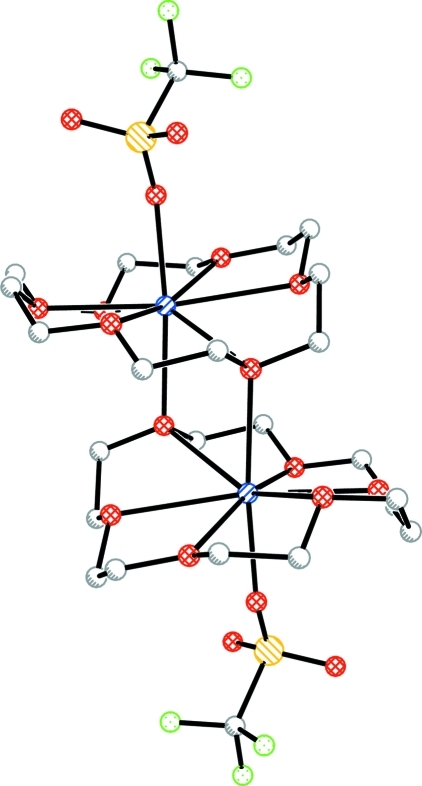
Partial packing diagram of the title compound showing the formation of a centrosymmetric dimer. Symmetry operator for generating equivalent atoms: 1 - x, 1 - y, 1 - z.

**Fig. 3.**
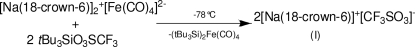
The preparation of the title compound.

## supplementary crystallographic information

### Comment

We report here the X-ray crystal structure analysis of sodium trifluorosulfonate as 18-crown-6 ether complex, [Na(18-crown-6)]^+^[CF_3_SO_3_]^-^. A huge number of hetereoleptic transition metal complexes with silyl ligands are known. Most of these compounds are synthesized by addition of silanes, *R*_3_SiH, to reactive transition metal species (Lerner, 2005). In contrast, few complexes with Fe—Si bonds have been structurally characterized. We have now investigated the reaction of Collman reagent Na_2_[Fe(CO)_4_] with *t*Bu_3_SiO_3_SCF_3_ (Lerner *et al.*, 2002). When [Na(18-crown-6)]_2_[Fe(CO)_4_] was treated with two molar equivalents of *t*Bu_3_SiO_3_SCF_3_, the title compound has been formed in nearly quantitative yield, as shown in the scheme below (scheme). The title compound, Na^+^[C_12_H_24_O_6_]^.^F_3_CSO_3_^-^, (Fig. 1) features a sodium cation that is coordinated by eight O atoms in an irregular hexagonal bipyramidal environment. The equatorial positions are occupied by the six O atoms of an 18-crown-6 ether ring with Na···O bond distances ranging from 2.5595 (17)Å to 3.0614 (18) Å. In the axial positions there is one O atom of a trifluoromethanesulfonate anion [Na1—O1S 2.3222 (18) Å] and an ether O atom of a symmetry equivalent crown-ether ring [Na1—O10^i^ 2.5792 (17) Å; symmetry operator (i): 1 - *x*, 1 - *y*, 1 - *z*]. In this way, centrosymmetric dimers are formed (Fig. 2).

For the structure of similar complexes with trifluoromethanesulfonate, see: Bolte & Lerner (2001); Lerner & Bolte (2003); Sofina *et al.* (2003); Dinnebier *et al.* (2004); Hildebrandt *et al.* (2006).

### Experimental

To a solution of Na_2_[Fe(CO)_4_] (75 mg, 0.35 mmol) and 18-crown-6 (92 mg, 0.35 mmol) in 20 ml of tetrahydrofuran was added a solution of *t*Bu_3_SiO_3_SCF_3_ (244 mg, 0.70 mmol) in 20 ml toluene at 195 K. The resulting orange-yellow solution was allowed to warm up to room temperature. Colourless crystals of the title compound were grown by storing this solution at room temperature for several days.

### Refinement

H atoms were refined with fixed individual displacement parameters [U(H) = 1.2 *U*_eq_(C)] using a riding model with C—H = 0.99 Å.

### Crystal data

**Table:**

[Na(CF_3_O_3_S)(C_12_H_24_O_6_)]	*F*(000) = 912
*M**_r_* = 436.37	*D*_x_ = 1.465 Mg m^−^^3^
Monoclinic, *P*2_1_/*n*	Mo *K*α radiation, λ = 0.71073 Å
Hall symbol: -P 2yn	Cell parameters from 9688 reflections
*a* = 9.4455 (9) Å	θ = 3.7–25.3°
*b* = 15.1723 (12) Å	µ = 0.26 mm^−^^1^
*c* = 14.0597 (14) Å	*T* = 173 K
β = 100.828 (8)°	Block, colourless
*V* = 1979.0 (3) Å^3^	0.33 × 0.20 × 0.19 mm
*Z* = 4	

### Data collection

**Table:**

Stoe IPDS II two-circle diffractometer	3697 independent reflections
Radiation source: fine-focus sealed tube	2856 reflections with *I* > 2σ(*I*)
graphite	*R*_int_ = 0.039
ω scans	θ_max_ = 25.6°, θ_min_ = 3.6°
Absorption correction: multi-scan (*MULABS*; Spek, 2009; Blessing, 1995)	*h* = −11→11
*T*_min_ = 0.921, *T*_max_ = 0.943	*k* = −18→16
11867 measured reflections	*l* = −17→17

### Refinement

**Table:**

Refinement on *F*^2^	Secondary atom site location: difference Fourier map
Least-squares matrix: full	Hydrogen site location: inferred from neighbouring sites
*R*[*F*^2^ > 2σ(*F*^2^)] = 0.044	H-atom parameters constrained
*wR*(*F*^2^) = 0.111	*w* = 1/[σ^2^(*F*_o_^2^) + (0.056*P*)^2^ + 0.8425*P*] where *P* = (*F*_o_^2^ + 2*F*_c_^2^)/3
*S* = 1.03	(Δ/σ)_max_ < 0.001
3697 reflections	Δρ_max_ = 0.55 e Å^−^^3^
245 parameters	Δρ_min_ = −0.44 e Å^−^^3^
0 restraints	Extinction correction: *SHELXL97* (Sheldrick, 2008), Fc^*^=kFc[1+0.001xFc^2^λ^3^/sin(2θ)]^-1/4^
Primary atom site location: structure-invariant direct methods	Extinction coefficient: 0.0078 (11)

### Special details

**Table:**

Geometry. All e.s.d.'s (except the e.s.d. in the dihedral angle between two l.s. planes) are estimated using the full covariance matrix. The cell e.s.d.'s are taken into account individually in the estimation of e.s.d.'s in distances, angles and torsion angles; correlations between e.s.d.'s in cell parameters are only used when they are defined by crystal symmetry. An approximate (isotropic) treatment of cell e.s.d.'s is used for estimating e.s.d.'s involving l.s. planes.
Refinement. Refinement of *F*^2^ against ALL reflections. The weighted *R*-factor *wR* and goodness of fit *S* are based on *F*^2^, conventional *R*-factors *R* are based on *F*, with *F* set to zero for negative *F*^2^. The threshold expression of *F*^2^ > σ(*F*^2^) is used only for calculating *R*-factors(gt) *etc*. and is not relevant to the choice of reflections for refinement. *R*-factors based on *F*^2^ are statistically about twice as large as those based on *F*, and *R*- factors based on ALL data will be even larger.

### Fractional atomic coordinates and isotropic or equivalent isotropic displacement parameters (Å^2^)

**Table:**

	*x*	*y*	*z*	*U*_iso_*/*U*_eq_	
S1	0.17698 (6)	0.56948 (4)	0.77270 (4)	0.02932 (18)	
O1S	0.30336 (19)	0.58449 (15)	0.73348 (12)	0.0471 (5)	
O2S	0.0687 (2)	0.63629 (17)	0.75273 (14)	0.0602 (6)	
O3S	0.1236 (3)	0.48077 (17)	0.76046 (16)	0.0800 (8)	
C1	0.2408 (3)	0.5785 (2)	0.90285 (18)	0.0451 (7)	
F1	0.3404 (2)	0.51872 (14)	0.93644 (11)	0.0652 (6)	
F2	0.2920 (3)	0.65607 (15)	0.92817 (15)	0.0950 (8)	
F3	0.1342 (2)	0.5651 (2)	0.95120 (13)	0.0976 (9)	
Na1	0.44188 (9)	0.58543 (6)	0.61340 (6)	0.0272 (2)	
O1	0.5534 (2)	0.75492 (12)	0.63049 (12)	0.0429 (5)	
C2	0.6072 (3)	0.7698 (2)	0.73050 (18)	0.0479 (7)	
H2A	0.5265	0.7701	0.7663	0.057*	
H2B	0.6555	0.8279	0.7394	0.057*	
C3	0.7129 (3)	0.6982 (2)	0.76966 (19)	0.0453 (7)	
H3A	0.7914	0.6957	0.7319	0.054*	
H3B	0.7560	0.7104	0.8382	0.054*	
O4	0.63713 (18)	0.61655 (12)	0.76219 (11)	0.0385 (4)	
C5	0.7169 (3)	0.54774 (19)	0.81833 (17)	0.0411 (7)	
H5A	0.7471	0.5669	0.8865	0.049*	
H5B	0.8043	0.5332	0.7921	0.049*	
C6	0.6208 (3)	0.46921 (18)	0.81295 (15)	0.0356 (6)	
H6A	0.6708	0.4208	0.8530	0.043*	
H6B	0.5323	0.4843	0.8376	0.043*	
O7	0.58458 (17)	0.44233 (11)	0.71374 (10)	0.0304 (4)	
C8	0.4890 (3)	0.36874 (16)	0.69957 (16)	0.0297 (5)	
H8A	0.3915	0.3867	0.7082	0.036*	
H8B	0.5242	0.3217	0.7469	0.036*	
C9	0.4847 (3)	0.33610 (15)	0.59799 (16)	0.0281 (5)	
H9A	0.5841	0.3240	0.5886	0.034*	
H9B	0.4299	0.2802	0.5885	0.034*	
O10	0.41817 (16)	0.39975 (10)	0.52663 (10)	0.0247 (3)	
C11	0.2659 (2)	0.38518 (16)	0.50056 (17)	0.0299 (5)	
H11A	0.2241	0.3796	0.5598	0.036*	
H11B	0.2471	0.3297	0.4632	0.036*	
C12	0.1974 (2)	0.46051 (17)	0.44098 (15)	0.0300 (5)	
H12A	0.2497	0.4724	0.3876	0.036*	
H12B	0.0962	0.4458	0.4124	0.036*	
O13	0.20166 (16)	0.53687 (11)	0.50144 (10)	0.0271 (4)	
C14	0.1175 (3)	0.60634 (18)	0.45126 (16)	0.0348 (6)	
H14A	0.0159	0.5872	0.4323	0.042*	
H14B	0.1539	0.6213	0.3917	0.042*	
C15	0.1263 (3)	0.68530 (18)	0.51541 (18)	0.0378 (6)	
H15A	0.0633	0.7328	0.4830	0.045*	
H15B	0.0946	0.6700	0.5766	0.045*	
O16	0.27303 (18)	0.71387 (11)	0.53489 (11)	0.0346 (4)	
C17	0.2986 (3)	0.79133 (18)	0.5926 (2)	0.0442 (7)	
H17A	0.2883	0.7786	0.6600	0.053*	
H17B	0.2289	0.8380	0.5663	0.053*	
C18	0.4496 (3)	0.82041 (17)	0.5897 (2)	0.0461 (7)	
H18A	0.4585	0.8318	0.5218	0.055*	
H18B	0.4701	0.8761	0.6263	0.055*	

### Atomic displacement parameters (Å^2^)

**Table:**

	*U*^11^	*U*^22^	*U*^33^	*U*^12^	*U*^13^	*U*^23^
S1	0.0269 (3)	0.0373 (4)	0.0243 (3)	−0.0015 (3)	0.0061 (2)	0.0048 (2)
O1S	0.0304 (10)	0.0817 (16)	0.0323 (9)	0.0052 (10)	0.0138 (7)	0.0117 (9)
O2S	0.0421 (12)	0.0902 (18)	0.0470 (11)	0.0338 (12)	0.0053 (9)	0.0125 (11)
O3S	0.116 (2)	0.0615 (16)	0.0580 (14)	−0.0449 (16)	0.0047 (13)	0.0051 (11)
C1	0.0474 (16)	0.0597 (19)	0.0290 (12)	0.0230 (15)	0.0091 (11)	−0.0047 (12)
F1	0.0718 (12)	0.0926 (15)	0.0305 (8)	0.0447 (11)	0.0077 (7)	0.0110 (8)
F2	0.132 (2)	0.0681 (15)	0.0672 (13)	0.0070 (14)	−0.0257 (13)	−0.0318 (11)
F3	0.0815 (15)	0.179 (3)	0.0427 (10)	0.0480 (16)	0.0394 (10)	0.0185 (12)
Na1	0.0237 (5)	0.0344 (5)	0.0246 (4)	0.0006 (4)	0.0070 (3)	0.0012 (4)
O1	0.0552 (12)	0.0341 (10)	0.0403 (10)	−0.0033 (9)	0.0115 (8)	−0.0154 (8)
C2	0.066 (2)	0.0401 (16)	0.0391 (14)	−0.0176 (15)	0.0135 (13)	−0.0194 (12)
C3	0.0407 (16)	0.0529 (18)	0.0414 (14)	−0.0238 (14)	0.0050 (11)	−0.0228 (13)
O4	0.0343 (10)	0.0438 (11)	0.0334 (9)	−0.0105 (8)	−0.0041 (7)	−0.0058 (8)
C5	0.0356 (14)	0.0611 (19)	0.0228 (11)	0.0009 (13)	−0.0047 (10)	−0.0059 (11)
C6	0.0392 (14)	0.0506 (16)	0.0162 (10)	0.0062 (12)	0.0031 (9)	0.0020 (10)
O7	0.0337 (9)	0.0384 (10)	0.0194 (7)	−0.0051 (7)	0.0055 (6)	0.0012 (6)
C8	0.0322 (13)	0.0283 (13)	0.0310 (12)	0.0021 (10)	0.0121 (9)	0.0083 (9)
C9	0.0294 (12)	0.0218 (12)	0.0345 (12)	0.0034 (10)	0.0101 (9)	0.0014 (9)
O10	0.0210 (8)	0.0252 (8)	0.0288 (8)	−0.0018 (6)	0.0067 (6)	0.0010 (6)
C11	0.0222 (12)	0.0322 (13)	0.0353 (12)	−0.0101 (10)	0.0052 (9)	−0.0049 (10)
C12	0.0232 (12)	0.0432 (15)	0.0222 (10)	−0.0071 (11)	0.0005 (8)	−0.0050 (9)
O13	0.0233 (8)	0.0359 (9)	0.0206 (7)	0.0048 (7)	0.0002 (6)	0.0046 (6)
C14	0.0225 (12)	0.0502 (16)	0.0304 (12)	0.0101 (11)	0.0020 (9)	0.0152 (11)
C15	0.0275 (13)	0.0443 (16)	0.0441 (14)	0.0162 (12)	0.0130 (10)	0.0163 (12)
O16	0.0358 (10)	0.0300 (10)	0.0425 (9)	0.0062 (8)	0.0185 (7)	0.0015 (7)
C17	0.0585 (19)	0.0302 (15)	0.0487 (15)	0.0136 (13)	0.0225 (13)	0.0000 (11)
C18	0.067 (2)	0.0215 (13)	0.0533 (16)	−0.0007 (13)	0.0213 (14)	−0.0059 (11)

### Geometric parameters (Å, °)

**Table:**

S1—O1S	1.4241 (18)	O7—C8	1.426 (3)
S1—O2S	1.430 (2)	C8—C9	1.505 (3)
S1—O3S	1.436 (2)	C8—H8A	0.9900
S1—C1	1.821 (3)	C8—H8B	0.9900
O1S—Na1	2.3222 (18)	C9—O10	1.448 (3)
C1—F2	1.296 (4)	C9—H9A	0.9900
C1—F1	1.328 (3)	C9—H9B	0.9900
C1—F3	1.333 (4)	O10—C11	1.433 (3)
Na1—O4	2.5595 (17)	O10—Na1^i^	2.5792 (17)
Na1—O10^i^	2.5792 (17)	C11—C12	1.491 (3)
Na1—O13	2.6129 (17)	C11—H11A	0.9900
Na1—O16	2.6265 (19)	C11—H11B	0.9900
Na1—O1	2.772 (2)	C12—O13	1.433 (3)
Na1—O7	2.7939 (19)	C12—H12A	0.9900
O1—C2	1.421 (3)	C12—H12B	0.9900
O1—C18	1.437 (3)	O13—C14	1.425 (3)
C2—C3	1.508 (4)	C14—C15	1.493 (4)
C2—H2A	0.9900	C14—H14A	0.9900
C2—H2B	0.9900	C14—H14B	0.9900
C3—O4	1.425 (3)	C15—O16	1.429 (3)
C3—H3A	0.9900	C15—H15A	0.9900
C3—H3B	0.9900	C15—H15B	0.9900
O4—C5	1.434 (3)	O16—C17	1.423 (3)
C5—C6	1.491 (4)	C17—C18	1.501 (4)
C5—H5A	0.9900	C17—H17A	0.9900
C5—H5B	0.9900	C17—H17B	0.9900
C6—O7	1.432 (3)	C18—H18A	0.9900
C6—H6A	0.9900	C18—H18B	0.9900
C6—H6B	0.9900		
			
O1S—S1—O2S	115.53 (13)	C5—C6—H6B	110.1
O1S—S1—O3S	113.85 (16)	H6A—C6—H6B	108.4
O2S—S1—O3S	114.78 (17)	C8—O7—C6	112.85 (17)
O1S—S1—C1	103.51 (12)	C8—O7—Na1	107.78 (12)
O2S—S1—C1	103.52 (12)	C6—O7—Na1	105.96 (14)
O3S—S1—C1	103.50 (14)	O7—C8—C9	107.10 (17)
S1—O1S—Na1	155.54 (12)	O7—C8—H8A	110.3
F2—C1—F1	108.7 (3)	C9—C8—H8A	110.3
F2—C1—F3	106.1 (3)	O7—C8—H8B	110.3
F1—C1—F3	105.5 (2)	C9—C8—H8B	110.3
F2—C1—S1	112.3 (2)	H8A—C8—H8B	108.5
F1—C1—S1	112.63 (18)	O10—C9—C8	111.66 (18)
F3—C1—S1	111.2 (2)	O10—C9—H9A	109.3
O1S—Na1—O4	79.91 (6)	C8—C9—H9A	109.3
O1S—Na1—O10^i^	174.33 (7)	O10—C9—H9B	109.3
O4—Na1—O10^i^	102.35 (6)	C8—C9—H9B	109.3
O1S—Na1—O13	83.81 (6)	H9A—C9—H9B	108.0
O4—Na1—O13	162.80 (6)	C11—O10—C9	110.99 (17)
O10^i^—Na1—O13	94.38 (5)	C11—O10—Na1^i^	116.86 (12)
O1S—Na1—O16	85.96 (7)	C9—O10—Na1^i^	111.66 (12)
O4—Na1—O16	119.60 (7)	O10—C11—C12	109.53 (18)
O10^i^—Na1—O16	88.42 (5)	O10—C11—H11A	109.8
O13—Na1—O16	64.28 (6)	C12—C11—H11A	109.8
O1S—Na1—O1	101.51 (7)	O10—C11—H11B	109.8
O4—Na1—O1	63.53 (6)	C12—C11—H11B	109.8
O10^i^—Na1—O1	75.19 (5)	H11A—C11—H11B	108.2
O13—Na1—O1	125.92 (6)	O13—C12—C11	109.00 (17)
O16—Na1—O1	62.54 (6)	O13—C12—H12A	109.9
O1S—Na1—O7	84.97 (6)	C11—C12—H12A	109.9
O4—Na1—O7	61.67 (6)	O13—C12—H12B	109.9
O10^i^—Na1—O7	100.68 (5)	C11—C12—H12B	109.9
O13—Na1—O7	111.53 (6)	H12A—C12—H12B	108.3
O16—Na1—O7	170.41 (6)	C14—O13—C12	110.70 (17)
O1—Na1—O7	122.53 (6)	C14—O13—Na1	115.48 (14)
C2—O1—C18	112.0 (2)	C12—O13—Na1	120.65 (13)
C2—O1—Na1	107.03 (15)	O13—C14—C15	109.39 (18)
C18—O1—Na1	112.78 (15)	O13—C14—H14A	109.8
O1—C2—C3	109.6 (2)	C15—C14—H14A	109.8
O1—C2—H2A	109.7	O13—C14—H14B	109.8
C3—C2—H2A	109.7	C15—C14—H14B	109.8
O1—C2—H2B	109.7	H14A—C14—H14B	108.2
C3—C2—H2B	109.7	O16—C15—C14	107.54 (19)
H2A—C2—H2B	108.2	O16—C15—H15A	110.2
O4—C3—C2	108.1 (2)	C14—C15—H15A	110.2
O4—C3—H3A	110.1	O16—C15—H15B	110.2
C2—C3—H3A	110.1	C14—C15—H15B	110.2
O4—C3—H3B	110.1	H15A—C15—H15B	108.5
C2—C3—H3B	110.1	C17—O16—C15	114.5 (2)
H3A—C3—H3B	108.4	C17—O16—Na1	110.39 (15)
C3—O4—C5	112.80 (19)	C15—O16—Na1	110.24 (14)
C3—O4—Na1	119.71 (15)	O16—C17—C18	106.7 (2)
C5—O4—Na1	122.61 (14)	O16—C17—H17A	110.4
O4—C5—C6	107.58 (19)	C18—C17—H17A	110.4
O4—C5—H5A	110.2	O16—C17—H17B	110.4
C6—C5—H5A	110.2	C18—C17—H17B	110.4
O4—C5—H5B	110.2	H17A—C17—H17B	108.6
C6—C5—H5B	110.2	O1—C18—C17	111.5 (2)
H5A—C5—H5B	108.5	O1—C18—H18A	109.3
O7—C6—C5	108.00 (18)	C17—C18—H18A	109.3
O7—C6—H6A	110.1	O1—C18—H18B	109.3
C5—C6—H6A	110.1	C17—C18—H18B	109.3
O7—C6—H6B	110.1	H18A—C18—H18B	108.0
			
O2S—S1—O1S—Na1	−80.8 (4)	O1S—Na1—O7—C8	71.26 (13)
O3S—S1—O1S—Na1	55.2 (4)	O4—Na1—O7—C8	152.50 (14)
C1—S1—O1S—Na1	166.8 (3)	O10^i^—Na1—O7—C8	−109.13 (12)
O1S—S1—C1—F2	61.9 (2)	O13—Na1—O7—C8	−10.05 (13)
O2S—S1—C1—F2	−59.0 (3)	O1—Na1—O7—C8	171.72 (12)
O3S—S1—C1—F2	−179.1 (2)	O1S—Na1—O7—C6	−49.80 (14)
O1S—S1—C1—F1	−61.2 (3)	O4—Na1—O7—C6	31.44 (13)
O2S—S1—C1—F1	177.9 (2)	O10^i^—Na1—O7—C6	129.81 (14)
O3S—S1—C1—F1	57.8 (3)	O13—Na1—O7—C6	−131.11 (14)
O1S—S1—C1—F3	−179.4 (2)	O1—Na1—O7—C6	50.66 (15)
O2S—S1—C1—F3	59.7 (3)	C6—O7—C8—C9	−168.50 (19)
O3S—S1—C1—F3	−60.4 (3)	Na1—O7—C8—C9	74.85 (18)
S1—O1S—Na1—O4	−169.0 (3)	O7—C8—C9—O10	−66.6 (2)
S1—O1S—Na1—O13	5.4 (3)	C8—C9—O10—C11	−90.6 (2)
S1—O1S—Na1—O16	70.0 (3)	C8—C9—O10—Na1^i^	137.13 (15)
S1—O1S—Na1—O1	130.9 (3)	C9—O10—C11—C12	170.05 (18)
S1—O1S—Na1—O7	−106.9 (3)	Na1^i^—O10—C11—C12	−60.3 (2)
O1S—Na1—O1—C2	47.64 (17)	O10—C11—C12—O13	−70.3 (2)
O4—Na1—O1—C2	−24.84 (16)	C11—C12—O13—C14	−170.82 (18)
O10^i^—Na1—O1—C2	−137.10 (17)	C11—C12—O13—Na1	49.8 (2)
O13—Na1—O1—C2	138.30 (16)	O1S—Na1—O13—C14	94.66 (15)
O16—Na1—O1—C2	126.87 (17)	O4—Na1—O13—C14	113.5 (2)
O7—Na1—O1—C2	−43.73 (18)	O10^i^—Na1—O13—C14	−79.95 (14)
O1S—Na1—O1—C18	−76.03 (16)	O16—Na1—O13—C14	6.18 (13)
O4—Na1—O1—C18	−148.50 (17)	O1—Na1—O13—C14	−5.08 (16)
O10^i^—Na1—O1—C18	99.24 (16)	O7—Na1—O13—C14	176.75 (13)
O13—Na1—O1—C18	14.64 (18)	O1S—Na1—O13—C12	−127.82 (15)
O16—Na1—O1—C18	3.21 (15)	O4—Na1—O13—C12	−108.9 (2)
O7—Na1—O1—C18	−167.39 (15)	O10^i^—Na1—O13—C12	57.58 (15)
C18—O1—C2—C3	178.8 (2)	O16—Na1—O13—C12	143.70 (16)
Na1—O1—C2—C3	54.7 (2)	O1—Na1—O13—C12	132.44 (14)
O1—C2—C3—O4	−63.7 (3)	O7—Na1—O13—C12	−45.72 (16)
C2—C3—O4—C5	−165.5 (2)	C12—O13—C14—C15	−178.97 (19)
C2—C3—O4—Na1	38.6 (3)	Na1—O13—C14—C15	−37.4 (2)
O1S—Na1—O4—C3	−116.58 (19)	O13—C14—C15—O16	63.5 (2)
O10^i^—Na1—O4—C3	58.12 (18)	C14—C15—O16—C17	177.68 (19)
O13—Na1—O4—C3	−135.6 (2)	C14—C15—O16—Na1	−57.14 (19)
O16—Na1—O4—C3	−37.1 (2)	O1S—Na1—O16—C17	69.56 (16)
O1—Na1—O4—C3	−8.22 (17)	O4—Na1—O16—C17	−6.43 (17)
O7—Na1—O4—C3	153.72 (19)	O10^i^—Na1—O16—C17	−109.75 (15)
O1S—Na1—O4—C5	89.95 (18)	O13—Na1—O16—C17	154.63 (16)
O10^i^—Na1—O4—C5	−95.35 (18)	O1S—Na1—O16—C15	−57.94 (15)
O13—Na1—O4—C5	70.9 (3)	O4—Na1—O16—C15	−133.92 (14)
O16—Na1—O4—C5	169.38 (16)	O10^i^—Na1—O16—C15	122.76 (14)
O1—Na1—O4—C5	−161.69 (19)	O13—Na1—O16—C15	27.13 (14)
O7—Na1—O4—C5	0.25 (16)	O1—Na1—O16—C15	−163.13 (15)
C3—O4—C5—C6	174.10 (19)	C15—O16—C17—C18	−169.6 (2)
Na1—O4—C5—C6	−30.8 (3)	Na1—O16—C17—C18	65.3 (2)
O4—C5—C6—O7	61.9 (3)	C2—O1—C18—C17	−93.2 (3)
C5—C6—O7—C8	−178.3 (2)	Na1—O1—C18—C17	27.7 (2)
C5—C6—O7—Na1	−60.6 (2)	O16—C17—C18—O1	−62.0 (3)

Symmetry codes: (i) −*x*+1, −*y*+1, −*z*+1.
